# Supplementary material for: Strain rate of stretch affects crossbridge detachment during relaxation of intact cardiac trabeculae
Source: PLoS One. 2024 Mar 4;19(3):e0297212. doi: 10.1371/journal.pone.0297212 (PMC10911597; doi:10.1371/journal.pone.0297212)
Supplement: Attachment — Submitted filename: tanner et al_response.pdf [file pone.0297212.s001.pdf]

Review Comments to the Author

We thank the reviewers for their comments regarding the manuscript. In addition to Editor requested items (Funding, Data), we have worked to correct typos, minor errors, and inconsistent labeling, and we have the discussion to enhance clarity.

Reviewer #1: The study aims to explore cross bridge mechanisms responsible for the lusitropic effect of small, fast, stretches in the twitching cardiac muscle. The subject may be of interest for both basic and translational research.

I had reviewed a previous version of this manuscript that had been rejected several months ago by a different journal. I must recognize that the changes the Authors have made to the present version of their work recognize most of the limits of the previous version. The changes are in line with the criticisms the Authors had received. The new version is improved even though the Authors should recognize, as a limitation of their work, that the stress vs time data calculated as the difference between the reference and ramp-stretch twitch traces (see Fig 1 central panel) are meaningful only if they assume that the Calcium transient and the myofilament activation level are unaffected by the applied stretches, i.e. the Authors are assuming that the activation and its decay are the same in the reference twitch and in the ramp-stretch twitch. The study provides some advancement in the description of the Mechanical Control of Relaxation reported in a previous paper.

> **RESPONSE**
> We thank the reviewer for their productive and positive comments. We have now expanded our discussion regarding the importance of time-varying calcium in a section called “Potential Impact of Time-varying Calcium Transients” at the end of the discussion. This section more carefully assesses how length changes might modify the calcium transient and modeling of the data. Specifically, multiple prior reports suggest that the impact within a twitch is minimal, whereas most changes would take minutes to develop and would not show up within the twitches that were analyzed in this study.

Minor points:
- the Authors should carefully check the text for typos and mistakes (e.g. Fig1 panels: the Authors should pay attention, there is no A B C panels in the Figure as indicated in the legend).
- ‘trabeculae’ is plural in Latin and ‘trabecula’ should be used for the singular case.

> **RESPONSE**
> We thank the reviewer for the careful review and comments. We have checked for errors and have carefully considered the singular or plural use of trabecula. Figure 1 has been corrected and labels in 1C added; we apologize for not catching this during the conversion of the figure for submission.

Reviewer #2: This is a manuscript which presents an elegant series of measurements on the effect of strain and strain rate on the relaxation of rat cardiac muscle fibers. The work is novel, technically challenging and well presented. It is a follow up to work published by the corresponding author in the J Mol Cell Cardiol in 2017 which established relaxation is dependent upon the rate of stretch just before the start of relaxation. In this study a potential confounding variable was tested (time to stretch and speed of stretch). - The conclusion is that all effects on the time of stretch were compatible with the view that cross bridge detachment and relaxation rate are accelerated by strain rate.

The work is sound and will be of interest to a specialist group of experimentalists.
The conclusions are sound but not very remarkable.

Minor issues
Introduction Line 13: “relaxaiton”
Methods: subscripts are missing for all chemical formulae e.g. KHCO3 - KHCO3.
Just before Fig 1 legend: “was acquired by repeating the load-clamp was repeated”
Fig 1 is missing A, B, C labels.
Fig 1C It is not clear what t12 refers to. It would be useful to include t0, t1 & t2
There are two Fig 6’s, one is Fig 5

RESPONSE
We thank the reviewer for the careful assessment. We have corrected the errors in the text and revised Figure 1 to include labels that were lost when converting to the format for submission.
